# A New Feature of Our Journal

**Published:** 1871-01

**Authors:** 


### A NEW FEATURE OF OUR JOURNAL.

At the suggestion of friends, we will add, in our next number, a separate and distinct department to our journal, to be appropriated to biographical sketches of living and deceased members of the med ical profession; to hygiene, and kindred subjects calculated to interest and instruct, both the professional and general reader. As far as practical, we will accompany each biographical sketch with a life-like engraving of the individual. To the professional reader, our biographical sketches will furnish a fund of valuable information, showing the trials, difficulties, vicissitudes and successes of eminent physicians, which may serve, not only as mementoes, but as distinguished landmarks, to point our young professional brethren to honor and distinction. To emulate the qualities of the great and good should be a part of the education of the young; and young physicians, by an intimate acquaintance with the lives of those who have attained to eminence in the profession, can more readily comprehend, and overcome, the difficulties besetting the pathway to professional success and honor.

This department thus devoted, as a means of information to all, will, we think, enhance the value of the Journal—particularly to the general, intelligent reader. To this class of readers we will endeavor to give original and selected articles, elucidating the principles or "laws" upon which true hygiene is founded; showing the importance of science in the prevention, as well as the management of, diseases; and the intimate relations which should exist, by virtue of scientific skill on the one hand, and trust and confidence on the other, between the profession and the public. To do this our object will be to break the charm of mystery with which the empiric invests himself; to strip him of his cloak of ignorance, and reveal him in his true colors, as an imposter.

Our energetic and accommodating publisher is making arrangements to carry into successful operation this new and important feature of the COMPANION.

